# Environmental co-benefits of a Mediterranean-style dietary intervention for reducing depressive symptoms in adults: results from the Curbing Anxiety and Depression using Lifestyle Medicine randomised controlled trial

**DOI:** 10.1017/S0007114525103942

**Published:** 2025-07-28

**Authors:** Megan Turner, Deborah N. Ashtree, Melissa M. Lane, Kim Anastasiou, Michalis Hadjikakou, Samantha L. Dawson, Mark Lawrence, Laura Jennings, Ozge Geyik, Felice N. Jacka, Vincent L. Versace, Mary Lou Chatterton, Pilvikki Absetz, Marita Bryan, Barbara Brayner, Sophie Mahoney, Dean Saunders, Tayla John, Lauren M. Young, Adrienne O’Neil

**Affiliations:** 1 Deakin University, IMPACT – The Institute for Mental and Physical Health and Clinical Translation, Food & Mood Centre, School of Medicine, Barwon Health, Geelong, Australia; 2 Deakin University, School of Psychology, 75 Pigdons Road, Waurn Ponds 3216, VIC, Australia; 3 Stretton Health Equity, Stretton Institute, School of Social Sciences, University of Adelaide, Adelaide 5000, SA, Australia; 4 Deakin University, Institute for Physical Activity and Nutrition, School of Exercise and Nutrition Sciences, Geelong, VIC 3220, Australia; 5 Centre for Integrative Ecology, School of Life and Environmental Sciences, Deakin University, Melbourne, VIC, Australia; 6 Deakin Rural Health, Deakin University, Princes Hwy, Warrnambool, VIC, Australia; 7 GeoHealth Labaratory, Department of Population Health, Dasman Diabetes Institute, Kuwait City, Kuwait; 8 School of Public Health and Preventative Medicine, Health Economics Group, Monash University, Melbourne, VIC, Australia; 9 Tampere University, Health Sciences, Faculty of Social Sciences, Kauppi Campus, Tampere, Finland; 10 Barwon Health MHDAS, 118 Moorabool Street, Geelong, VIC 3220, Australia

**Keywords:** Mediterranean diet, Environmental sustainability, Lifestyle therapy, Sustainable diets, Nutritional psychiatry

## Abstract

This study explored whether lifestyle therapy that promoted adherence to a Mediterranean-style diet as a treatment for depression led to environmental co-benefits. Participants (*n* 75 complete case) were Australian adults in the Curbing Anxiety and Depression using Lifestyle Medicine non-inferiority, randomised controlled trial, which showed that lifestyle therapy was non-inferior to psychotherapy in reducing depressive symptoms, when delivered in group format via video conferencing over an 8-week treatment period. In this secondary analysis, we hypothesised that the lifestyle arm would be superior to the psychotherapy arm in reducing the environmental impact of self-reported diet over time. Dietary intake derived from FFQ at baseline and 8 weeks was transformed into environmental impact scores by calculating global warming potential (GWP)*. GWP* was calculated for total dietary intake and distinct food groups (Australian Dietary Guidelines and NOVA classifications). Within-arm changes in GWP* over time were calculated using the median difference. Neither arm showed significant changes. Between-arm differences in percentage change in GWP* scores over time were analysed using generalised estimating equations models. No between-arm difference for total GWP* score was found (*β* = 11·06 (–7·04, 29·15)). When examining distinct food groups, results were mixed. These novel findings contribute to the sparse evidence base that has measured the environmental impact of diets in a clinical trial context. Whilst lifestyle therapy that reduced depressive symptoms did not have clear environmental benefits relative to psychotherapy, nutritional counselling that focuses on the environmental impact of food choices may drive more pronounced planetary co-benefits.

Lifestyle therapy targets health behaviours such as nutrition and physical activity and is a clinically and cost-effective approach to managing mental disorders^(1–3)^. The field of nutritional psychiatry has provided extensive evidence of independent associations between diet quality and depression risk^(4)^. There is also clinical trial evidence that Mediterranean-style dietary interventions, which promote higher intake of whole foods such as fresh fruit and vegetables, wholegrains and legumes, can reduce mental health symptoms in those with major depressive disorder when delivered under the supervision of an Accredited Practising Dietitian^(5,6)^. Recently, the Curbing Anxiety and Depression using Lifestyle Medicine (CALM) non-inferiority trial found that a lifestyle treatment produced comparable reductions in depressive symptoms over an 8-week period to psychotherapy^(7)^. Importantly, participants in the lifestyle arm showed higher adherence to a Mediterranean-style diet and reported reductions in the percentage of food intake from discretionary items compared with those in the psychotherapy arm at 8 weeks; however, no between-arm differences were observed for physical activity^(7)^.

From an environmental perspective, diets higher in fresh, plant-based foods have been associated with lower greenhouse gas emissions (GHGe)^(8,9)^ and subsequent environmental impacts^(10,11)^. Thus, given that food production accounts for approximately one-third of total global GHGe, there may be environmental co-benefits to using a Mediterranean-style diet to prevent and treat mental health symptoms^(12–14)^. Whilst cross-sectional studies suggest a link between dietary intake and environmental impacts, there is limited data from clinical trials^(15)^, and there are even fewer clinical trials that have explored the intersection of diet, mental health and the environment.

A 2019 study – a secondary analysis of the Mood Food trial – examined the environmental impact of a Mediterranean diet intervention for 744 people who were overweight and experiencing subsyndromal depression at 6 and 12 months^(16)^. Using a life cycle assessment with an aggregate indicator of GHGe, land use and fossil energy use as the primary outcome, the authors found no evidence that a Mediterranean diet resulted in a reduced environmental footprint^(16)^. In contrast, a 2024 study found that higher adherence to a Mediterranean-style diet, which was designed to reduce metabolic risk in overweight adults in Spain, was associated with decreased environmental impact when controlling for energy intake and found that GHGe were reduced if fish consumption was eliminated^(15)^. Thus, further scrutiny of the environmental impacts of a Mediterranean-style diet, particularly GHGe, is warranted.

The aim of this study was to compare changes in global warming potential (GWP)* – as a measure of climate impact of emitted greenhouse gases (GHGe) – of self-reported diets of participants engaging in a lifestyle therapy focused on nutritional and exercise counselling, relative to psychotherapy, using data from the CALM randomised controlled trial^(7,17)^. We hypothesised that, from baseline to 8 weeks, participants in the lifestyle arm would show significantly greater reductions in GHGe (measured by GWP*) based on self-reported dietary intake, relative to the psychotherapy arm.

## Methods

The present study is reported using the Consolidated Standards of Reporting Trials (CONSORT) statement for parallel-group randomised trials^(18)^. The original CALM trial protocol has been published previously^(17)^ and was registered through the Australian and New Zealand Clinical Trials Registry (trial ID: ACTRN12621000387820). The analysis plan for the present study was registered on the Open Science Framework in 2023 (·https://doi.org/10.17605/OSF.IO/JQP6R).

### Design

CALM is a two-arm, parallel-group, individually randomised group treatment, non-inferiority trial that demonstrated the effectiveness of lifestyle therapy (*n* 70) for reducing depressive symptoms, compared with an active control group (psychotherapy; *n* 62)^(7,17)^. For the present study, treatment arms were compared on their environmental impact (measured by percentage change in GWP*) calculated from self-reported dietary data collected at baseline and 8 weeks.

### Treatment arms

Both treatments have been described in detail previously^(7,17)^. Briefly, treatment was remotely delivered using videoconferencing, with communication between interventionists and participants otherwise occurring over telephone or email. Both treatments were manualised for standardisation and comprised six group-based sessions (4–10 people) over 8 weeks.

#### Lifestyle therapy

The programme was focused on promoting adherence to a Mediterranean-style diet and increased physical activity. The Mediterranean-style diet was informed by previous models, including the ModiMed diet^(19)^, the Finnish Diabetes Prevention Study^(20)^, the GOAL programme^(21)^ and the Australian Greater Green Triangle Diabetes Prevention Project^(22)^. The three goals for programme participants were (1) a maximum of 10 % of dietary energy from saturated fat, (2) a minimum of 15 g/1000 kcal fibre and (3) a minimum of 150 min/moderate physical activity or 75 min/vigorous physical activity per week.

#### Psychotherapy

A transdiagnostic group cognitive behaviour therapy programme was developed and delivered by psychologists for use as an active control condition. It was adapted from the Mood Management course developed by the Centre of Clinical Interventions^(23)^. The aims of the programme were to develop skills in self-awareness and identifying and managing unhelpful thoughts and behaviours using strategies such as cognitive disputation and behavioural experiments.

### Participants and procedures

The CALM trial recruited participants aged 18 years or older, residing in Victoria, Australia, who could provide informed consent, communicate in English and who could commit to attending six group sessions online. Participants with indicative depression (a Distress Questionnaire-5^(24)^ equal to or greater than 8) at enrolment were eligible to participate. Exclusion criteria included a known or suspected clinically unstable medical or mental health disorder (including acute suicidality), current or lapsed eating disorder, currently pregnant or breastfeeding and starting a new treatment within 1 month prior to their baseline assessment. Participants were recruited from a tertiary mental health service in Geelong, Australia, as well as through community-based advertising. The service predominately sees patients from the metropolitan area (Modified Monash 1, MM1) of Geelong (the main population centre) but also services regional centres (MM2), large rural towns (MM3), medium rural towns (MM4) and small rural towns (MM5)^(25)^. Interventions were delivered between August 2020 and April 2022.

#### Environmental impact assessment

Environmental impacts of food systems are complex and can include several key environmental indicators such as water use, land use, fertiliser and pesticide use and GHGe^(26,27)^. The GWP* was chosen in this study because it has a reliable, comparative dietary dataset tested in an Australian sample^(28)^ and better represents the warming effects of short-lived climate pollutants like methane, by considering both emission rates and cumulative impact. That is, GWP* provides a nuanced view of how short-lived pollutants contribute to global warming^(29,30)^. Higher values indicate higher warming potential and thus worse environmental impacts. Based on their study of 9341 Australian adults, Ridoutt *et al.* found that the climate footprint of the average current diet was highest for fresh meat and alternatives (1·23 kg CO_2_-e daily per person) and discretionary choices (1·15 kg CO_2_-e daily per person), whereas the climate footprint of the average current diet was lowest for fruit (0·12 kg CO_2_-e daily per person) and vegetables (0·15 kg CO_2_-e daily per person)^(28)^.

To generate an environmental impact score using the GWP* for each participant, we used self-reported dietary data at baseline and 8-week follow-up, collected using a validated FFQ (DQES v3.2) for Australian populations^(31,32)^. Next, the environmental impacts of individual foods were calculated using the GWP*. For this study, GWP* scores for all raw food variables were based on calculations from a previous Australian study, where the explicit calculation parameters are outlined with reference to the Australian Dietary Guidelines (ADG)^(28)^ (see ·https://www.mdpi.com/article/10.3390/nu13041122/s1 for details). GWP* results are reported as an environmental impact score in the unit kg/CO_2_-e (equivalent). To calculate GWP* for this study, using Ridoutt *et al.*’s dataset^(28)^, author LJ catalogued individual food items, categorised according to the ADG^(33)^ alongside their corresponding environmental factor codes (see online Supplementary Table 1). Next, authors LJ and KA aligned these food items with their dietary counterparts from the FFQ. In cases where a food item included more than one component or was ambiguous, we disaggregated the item and assigned percentage codes to best represent environmental scores, with agreement across three authors required (LJ, KA, MH) (e.g. 20 % olive oil and 80 % vegetables for the DQES V3.2 food item: ‘Oil on vegetables’).

As a novel concept in nutrition research, we also applied the NOVA food classification system to the dietary data to explore the environmental impact of different levels of industrial food processing^(34,35)^. NOVA classifications are (1) unprocessed or minimally processed (e.g. fresh produce, rice), (2) processed culinary ingredients (e.g. butter, honey, oils), (3) processed foods (e.g. canned vegetables, cheese, beer and wine) and (4) ultra-processed foods and drink products (UPF) (e.g. industrial formulations with five or more ingredients, such as distilled alcoholic beverages, ice cream and packaged snacks). The GWP* applied according to these categories is also displayed in online Supplementary Table 1.

### Data analysis

Based on data from Ridoutt *et al.* ^(28)^, the total daily GWP* score for the average Australian diet is 3·53 kg/CO_2_-e, and the total GWP* for a more healthful diet is 2·07 kg/CO_2_-e. We modelled power based on estimated group sample sizes of 68 and 75, a one-sided alpha of 0·05 and a range of standard deviations between 0·25 and 4·0. Based on our sample size, the study was sufficiently powered for results with standard deviations of 3·0 or less.

First, we investigated within-arm changes in reported diet and GWP* scores between baseline and 8-week endpoint. Next, a generalised estimating equations (GEE) model was fit to assess the impact of treatment arm (lifestyle *v*. psychotherapy) on percentage change in total GWP* scores from baseline to 8 weeks, accounting for the correlation due to the group-based intervention approach. The psychotherapy arm was set as the reference group. GEE analyses were adjusted for energy intake using Willett’s residual method (continuous)^(36)^ given the likely impact on outcomes^(15)^ and biological sex (binary) given the baseline imbalance between the two groups. The GEE model incorporated repeated measures from both baseline and 8-week time points. Identity for linear GEE was applied as a link function, and an exchangeable correlation structure was selected to appropriately account for the correlations within repeated measures. Results are presented as *β*-coefficients and corresponding 95 % CI and *P*-values. Significant findings were considered those with *P*-values < 0·05.

Analyses were run as complete cases (lifestyle arm (*n* 35), psychotherapy arm (*n* 39)), with secondary intention-to-treat (lifestyle arm (*n* 91), psychotherapy arm (*n* 91)) and per-protocol (lifestyle arm (*n* 71), psychotherapy arm (*n* 64)) models to explore the impact of missing data on the results. We adjusted for multiple comparisons using the Simes method.

Given the differential environmental impact of distinct food groups^(11,28,37)^ and the within-arm changes in median food intake and GWP* shown for lifestyle participants, we undertook *post hoc* exploratory analyses to explore the impact of individual food classifications on GWP* using both the ADG and NOVA classification systems.

## Results

The CONSORT flowchart was reported in the main outcome paper^(7)^ (··–https://www.thelancet.com/cms/10.1016/j.lanwpc.2024/101142/asset/ff5bacc6-2cdc-48e6-bcc5-a1cbbdc86cf9/main.assets/gr2_lrg.jpg). In the present study, we report data for the complete case sample (*n* 75; individuals with full dietary data available at baseline and follow-up), in addition to the intention-to-treat and per-protocol samples, with the latter two groups illustrated on the flowchart.

Participants were predominantly female, aged in their mid-to-late 40s and employed, and baseline mean depression scores (Patient Health Questionnaire-9) indicated mild-moderate depression. More male participants were assigned to the psychotherapy arm, but treatment arms did not differ on other demographic measures (see Table 1).

**Table 1.** Demographic characteristics mean (standard deviation) or frequency (%) of participants at baseline

	Total		Psychotherapy (*n* 64)		Lifestyle therapy (*n* 71)	
	Mean	sd	Mean	sd	Mean	sd
Participant age	45·3	14·2	43·9	13·8	46·6	14·4
	Frequency	%	Frequency	%	Frequency	%
Birth sex						
Male	20	14·8 %	15	23·4 %	5	7·0 %
Female	115	85·2 %	49	76·6 %	66	93·0 %
Born in Australia	111	82·8 %	50	79·4 %	61	85·9 %
Education						
Completed high school	120	88·9 %	59	92·2 %	61	85·9 %
Not completed high school	15	11·1 %	5	7·8 %	10	14·1 %
Employment status						
Employed	98	72·6 %	45	70·3 %	53	74·6 %
	Mean	sd	Mean	sd	Mean	sd
SEIFA	1020·3	50·8	1029·2	45·8	1012·7	54·2
BMI kg/m^2^	27·8	6·4	27·4	6·4	28·2	6·5
MEDAS score	4·7	1·8	4·9	1·8	4·5	1·8
Physical activity (total active hours)	4·1	3·0	3·8	2·8	4·3	3·1
Total PHQ-9 at baseline	10·3	5·8	10·4	5·4	10·1	6·1

SEIFA, Socioeconomic Index for Areas, Index of Relative Socioeconomic Disadvantage; PHQ, Patient Health Questionnaire-9; MEDAS, Mediterranean Diet Adherence Screener.

### Within-arm outcomes

Online Supplementary Table 2 shows the median dietary intake by treatment arm, mapped against the ADG^(33)^ and NOVA classifications^(35)^ at both timepoints. This shows that participants in the lifestyle arm reduced their discretionary food intake (and animal-source discretionary food) and increased their fish/seafood and vegetable consumption between baseline and 8 weeks. Those in the psychotherapy arm did not show statistically significant change in their dietary intakes in any food groups.

Table 2 shows the median baseline and 8-week GWP* and median difference over time for each arm. Neither group showed significant changes in total GWP* over time. Both treatment arms showed higher median GWP* at baseline and at 8 weeks than the current diets of Australian adults (3·53 kg CO_2_-e daily per person) reported by Ridoutt *et al.* ^(28)^. Psychotherapy participants had a median total GWP* of 5·11 (2·84–6·49) at baseline and 4·39 (2·57–5·91) at 8 weeks. Lifestyle participants had a median total GWP* of 4·66 (3·27–6·83) at baseline and 5·20 (3·75–6·17) at 8 weeks. The psychotherapy arm showed no significant median difference in GWP* over time for any food classification groups. The lifestyle arm showed significant median differences in the following ADG food classifications: discretionary foods (–0·10 (–0·21, –0·02)), animal-source discretionary foods (–0·07 (–0·13, –0·01)), fish/seafood (0·03(0·01, 0·06)) and vegetables (0·02(0·005, 0·031)). Using the NOVA classifications, the lifestyle arm showed a significant median difference on the ultra-processed food category (–0·07(–0·15, –0·00)). That is, lifestyle participants showed lower median GWP* scores in discretionary foods and UPF and higher median GWP* scores for fish/seafood and vegetables, at 8 weeks compared with baseline.

**Table 2.** Median (quartile 1 to quartile 3) baseline and 8-week follow-up GWP* score; median difference and 95 % confidence intervals (baseline to 8 weeks)

	Psychotherapy (*n* 64)						Lifestyle therapy (*n* 71)					
	Baseline median	q1–q3	8 weeks median	q1–q3	Median difference^†^	95 % CI	Baseline median	q1–q3	8 weeks median	q1–q3	Median difference^†^	95 % CI
NHMRC/ADG groups												
Total	5·11	2·84–6·39	4·39	2·57–5·91	–0·62	–1·46, 0·10	4·66	3·27–6·83	5·20	3·75–6·17	0·12	–0·67, 0·91
Dairy product and alternatives	0·39	0·18–0·63	0·35	0·19–0·55	–0·03	–0·13, 0·07	0·39	0·27–0·64	0·48	0·31–0·60	0·04	–0·07, 0·14
Dairy products	0·39	0·18–0·63	0·35	0·18–0·55	–0·03	–0·13, 0·07	0·39	0·27–0·64	0·48	0·31–0·59	0·04	–0·07, 0·14
Dairy product alternative	0·00	0·00–0·00	0·00	–0·00–0·00	0·00	0·00, 0·00	0·00	0·00–0·00	0·00	0·00–0·00	0·00	0·00, 0·00
Discretionary	0·40	0·23–0·58	0·37	0·25–0·52	–0·02	–0·12, 0·06	0·43	0·28–0·75	0·39	0·23–0·55	***–0·10***	***–0·21, −0·02***
Animal source	0·22	0·15–0·41	0·24	0·14–0·33	–0·01	–0·07, 0·04	0·27	0·18–0·47	0·23	0·14–0·34	***–0·07***	***–0·13, −0·01***
Other	0·14	0·08–0·24	0·12	0·07–0·21	–0·02	–0·06, 0·02	0·14	0·08–0·31	0·11	0·06–0·23	–0·03	–0·07, 0·01
Fat	0·04	0·03–0·06	0·05	0·03–0·06	0·003	–0·06, 0·011	0·05	0·03–0·08	0·06	0·04–0·09	0·01	–0·00, 0·02
Fruit	0·09	0·06–0·12	0·09	0·05–0·14	0·00	–0·02, 0·02	0·09	0·05–0·13	0·10	0·07–0·14	0·003	–0·02, 0·03
Grains	0·08	0·03–0·11	0·08	0·04–0·12	0·003	–0·002, 0·003	0·09	0·05–0·13	0·09	0·05–0·13	0·002	–0·02, 0·03
Lean meats and alternatives	0·79	0·42–1·22	0·66	0·44–1·16	–0·08	–0·24, 0·11	0·70	0·40–1·21	0·81	0·49–1·27	0·06	–0·14, 0·25
Eggs	0·03	0·02–0·07	0·03	0·03–0·07	0·00	0·00, 0·00	0·03	0·03–0·07	0·03	0·03–0·07	0·00	0·00, 0·00
Fish/seafood	0·07	0·03–0·12	0·07	0·03–0·13	–0·001	–0·03, 0·02	0·05	0·03–0·08	0·10	0·05–0·20	***0·03***	***0·01, 0·06***
Meat alternatives	0·04	0·02–0·09	0·04	0·02–0·08	0·00	–0·01, 0·01	0·05	0·03–0·08	0·06	0·04–0·10	0·02	–0·00, 0·03
Monogastric meat	0·16	0·09–0·27	0·14	0·07–0·23	–0·01	–0·06, 0·03	0·15	0·09–0·23	0·14	0·08–0·23	–0·00	–0·05, 0·03
Ruminant meat	0·37	0·07–0·50	0·31	0·01–0·87	–0·02	–0·19, 0·06	0·31	0·05–0·87	0·44	0·07–0·87	0·00	–0·12, 0·18
Vegetables	0·06	0·04–0·09	0·06	0·05–0·08	0·01	–0·008, 0·02	0·05	0·03–0·07	0·07	0·05–0·09	***0·02***	***0·005, 0·031***
Water-based beverages	2·34	0·83–4·23	2·23	0·81–3·99	–0·31	–1·24, 0·77	2·29	1·32–4·17	2·54	1·60–4·18	0·06	–0·48, 0·68
Nova classifications												
Unprocessed/minimally processed	4·18	2·24–5·65	3·90	2·51–5·61	–0·52	–1·29, 0·19	3·53	2·09–5·19	4·07	2·95–5·48	0·16	–0·62, 0·89
Processed culinary ingredients	0·07	0·05–0·12	0·08	0·06–0·15	0·00	–0·02, 0·02	0·07	0·05–0·12	0·09	0·06–0·16	0·00	–0·02, 0·03
Processed foods	0·21	0·13–0·31	0·28	0·11–0·41	–0·04	–0·10, 0·01	0·17	0·12·–0·25	0·28	0·15–0·42	–0·00	–0·07, 0·07
Ultra-processed foods	0·29	0·19–0·46	0·32	0·22–0·62	–0·03	–0·10, 0·03	0·29	0·19–0·39	0·26	0·205–0·44	***–0·07***	***–0·15, −0·00***

GWP*, global warming potential*; NHMRC/ADG, National Health and Medical Research Council/Australian Dietary Guidelines. Bold/italics indicates statistical significance (*P* < .05).

† Hodges-Lehmann median difference.

### Main outcome

We found that the percentage change in GWP* score over time did not differ between treatment arms (*β* = 11·06 (95 % CI −7·04, 29·15)) (see Table 3). These results were replicated using per-protocol and intention-to-treat samples (see online Supplementary Table 3).

**Table 3.** Association of treatment arm with % change in GWP* score between baseline and 8 weeks, adjusting for group participation, using complete case^†^ generalised estimating equations models^‡^

		*β*	L95CI, U95CI	*P*-value^§^	q-value^¶^
Total GWP*		11·06	–7·04, 29·15	0·116	0·226
NHMRC/ADG groups					
Dairy products and alt.		33·83	7·27, 60·40	**0·007**	0·058
	Dairy products	14·21	–38·64, 67·06	0·299	0·329
	Dairy product alt.	129·23	19·43, 239·02	**0·011**	0·058
Discretionary		–13·81	–28·53, 0·92	**0·033**	0·113
	Animal source	–7·76	–30·77, 15·24	0·254	0·294
	Other	–11·33	–37·80, 15·14	0·201	0·276
Unsaturated spreads and oils		32·40	8·83, 55·97	**0·004**	0·058
Fruit		12·30	–15·48, 40·07	0·193	0·276
Grains		68·32	–275·65, 412·29	0·349	0·365
Lean meats and alternatives		16·96	–12·97, 46·89	0·134	0·226
	Eggs	21·98	–11·75, 55·72	0·101	0·226
	Fish/seafood	76·13	0·76, 151·51	**0·024**	0·106
	Meat alternatives	77·89	14·09, 141·70	**0·009**	0·058
	Monogastric meat	8·83	–6·48, 24·15	0·129	0·226
	Ruminant meat	40·11	–66·90, 147·12	0·232	0·283
Vegetables		10·85	–15·93, 37·63	0·214	0·276
Water-based beverages		2·28	–31·22, 35·77	0·447	0·447
NOVA classifications					
Unprocessed/minimally processed		10·98	–7·30, 29·25	0·120	0·226
Processed culinary ingredients		13·26	–13·28, 39·79	0·164	0·257
Processed foods		17·78	–1·61, 37·17	**0·036**	0·113
Ultra-processed foods		–10·71	–23·28, 1·86	**0·048**	0·131

GWP*, global warming potential*; NHMRC/ADG, National Health and Medical Research Council/Australian Dietary Guidelines. Boldface indicates statistical signifcance (*P* < .05).

† Complete case analysis (*n* 75) with complete dietary data at baseline and 8 weeks.

‡ Adjusted for biological sex and energy intake using Willett’s residual method (36).

§ One-sided *P*-value for superiority hypothesis.

¶ Simes adjusted q-value (adjusting for multiple testing).

### *Post hoc* analyses

*Post hoc* analyses of individual food classifications are shown in Table 3. The adjusted GEE models found that percentage change in GWP* score over time increased in the lifestyle arm relative to psychotherapy for the following ADG categories: dairy products and alternatives (*β* = 33·83 (95 % CI 7·27, 60·40)), dairy product alternatives (*β* = 129·23 (95 % CI 19·43, 239·02)), unsaturated spreads and oils (*β* = 32·40 (95 % CI 8·83, 55·97)), fish/seafood (*β* = 76·13 (95 % CI 0·76, 151·51)) and meat alternatives (*β* = 77·89 (95 % CI 14·09, 141·70)). Likewise, we found weak evidence that GWP* may have increased for the lifestyle arm relative to psychotherapy for the NOVA processed food category (*β* = 17·78 (95 % CI −1·61, 37·17)). That is, there may have been an increased negative environmental impact as measured by the GWP* for the lifestyle arm from baseline to post-treatment, when looking at these specific food groups.

In contrast, the percentage change in GWP* score between baseline and 8 weeks decreased in the lifestyle arm relative to psychotherapy for the ADG category of discretionary foods (*β* = −13·81 (–28·53, 0·92)) and NOVA classification of UPF (*β* = −10·71 (–23·28, 1·86)). This suggests a decreased negative environmental impact from these classifications of food following lifestyle therapy.

There were no treatment arm differences in GWP* on the remaining food groups. The strength of these results was reduced after adjusting for multiple testing (see q-scores in Table 3) and when using per-protocol and intention-to-treat models (see online Supplementary Table 3), indicating that some of the ‘significant’ differences may have been due to the increased likelihood of false positives from multiple comparisons or due to the impact of missing data.

## Discussion

This study investigated whether a lifestyle intervention that promoted adherence to a Mediterranean-style diet and reduced depressive symptoms at a comparable magnitude as psychotherapy had incidental environmental co-benefits. We found no evidence that total GWP* (kg/CO_2_-e per person per day), as a measure of environmental impact, was reduced following participation in a lifestyle therapy when compared with psychotherapy. Thus, our main hypothesis was not supported. This finding is consistent with a secondary analysis of another clinical trial, which found no evidence that adherence to a Mediterranean diet in people experiencing depression reduced environmental impact^(16)^. However, these findings contrast with other research showing that adherence to a Mediterranean diet is associated with the lowest GHGe, land use and water use^(10,11)^. These discrepancies may be due to differences in study design, population characteristics or the specific environmental metrics assessed, suggesting that more research is needed to fully understand the environmental co-benefits of dietary interventions like the Mediterranean diet.

Within-arm analysis of median differences suggested that for the lifestyle arm only, food intake decreased in distinct categories, namely, discretionary foods and animal-source discretionary foods (ADG classifications) and UPF (NOVA classification). Further, the lifestyle arm increased their intake of fish/seafood and vegetables (ADG classifications). *Post hoc* analyses of between-arm differences in GWP* found increases in some ADG categories (dairy products and alternatives, dairy product alternatives, unsaturated spreads and oils, fish/seafood, meat alternatives) and decreases in others (discretionary items classified according to ADG and UPF (NOVA classification)) for the lifestyle arm relative to psychotherapy. In contrast, psychotherapy participants did not show significant median differences across any food groups for intake or GWP* scores.

These *post hoc* analyses offer a potential explanation for the null finding in the present study, as the results for the lifestyle arm suggested changes in their dietary patterns, with increases in some food categories and decreases in others, which may have contributed to an overall unchanged GWP* score. That is, increased intake and GWP* output in some areas may have ‘cancelled out’ reductions in others. It is also worth noting that using the ADG classification, the highest intake and GWP* score in both treatment arms came from water-based beverages, which included coffee, coffee substitutes, tea and herbal teas. The absence of significant reductions in energy intake or GWP* scores between baseline and 8 weeks in this category may have adversely impacted the feasibility of meaningful change in the total GWP* score. Replacing coffee and tea intake with water might be an appropriate nutritional recommendation for a lifestyle therapy to maximise the human and environmental benefits of the intervention.

The finding of reduced GWP* from discretionary items (ADG) and UPF (NOVA) in the lifestyle arm also warrants further consideration. Alcoholic beverages are included under discretionary items and could be a modifiable target of lifestyle therapy if broadened to include a focus on alcohol and substance use, consistent with the evidence base for lifestyle-based treatments of depression^(3)^. Furthermore, given the robust association between UPF dietary patterns and mental health^(38)^ and their adverse (and likely underreported) environmental impacts^(39,40)^, supporting people to focus on nutritious and low-processed food swaps might be an inexpensive addition to lifestyle therapy in a mental health context^(41)^. For example, ‘label reading’ was included as part of the nutritional counselling in the CALM trial^(17)^. This could be expanded and reinforced in future lifestyle therapy programmes, with reference to specific food classifications such as NOVA. A task such as counting the number of ingredients on a packet might be a practicable way of determining whether a food item is ultra-processed and support participants to make informed food choices with respect to their own health and the health of the planet. Furthermore, understanding the environmental impacts of different food groups, including discretionary foods such as processed meats, might support adherence to dietary changes, particularly for people who are concerned or anxious about the environment and climate change or have altruistic values. For instance, cross-sectional data have shown a direct relationship between pro-environmental behaviour, healthy behaviour and dietary adherence^(42,43)^. Given increasing evidence of a link between climate change and adverse mental health impacts^(44)^, lifestyle therapy that includes nutritional counselling promoting both human and planetary health is another potential avenue for treatment approaches in this developing field^(45)^.

Finally, given the relatively higher proportion of GHGe attributed to animal-based products such as dairy products and meats^(11,37)^, future iterations of Mediterranean-style diets for mental health might benefit from the inclusion of specific and localised recommendations around sustainable animal protein, such as poultry, eggs and fish and seafood. However, this needs to be balanced with the health consequences, given meta-analytic evidence of a direct relationship between meat consumption and mental health^(46)^ and the potential nutritional consequences of reducing animal-sourced foods^(47)^. Promisingly, the EAT-Lancet planetary health diet has been developed with the intention of maximising both human and planetary health, and it promotes consumption of vegetables, greens, fruits and wholegrains, with reduced consumption of meat, fish, eggs, refined cereals and tubers^(48)^. A recent cross-sectional study suggests that higher adherence to the planetary health diet is associated with a lower risk of anxiety and depression^(49)^. Future research that compares different dietary approaches for both their mental health and environmental impacts might be helpful in nuancing dietary guidelines to ensure a balanced and evidence-based approach that is also culturally responsive and locally relevant. The EAT-Lancet planetary health diet is currently undergoing a second iteration, but to our knowledge, this does not include any indicators for psychological outcomes, which we hope might be addressed through further research or in future iterations.

Whilst we believe that our findings have made a novel contribution to the relatively sparse evidence base that has measured the environmental impact of diets in a clinical trial context, this study is not without limitations. Whilst the initial power calculations demonstrated that this study was adequately powered to detect differences, a relatively smaller proportion provided complete dietary data at baseline and 8 weeks; hence, the complete case sample size was much smaller than predicted and therefore may have been underpowered to detect differences. This is supported by the reduced strength of our findings when using the intention-to-treat (randomised) and per-protocol (treatment completers) samples. Future studies might benefit from further consideration of how to promote adherence to dietary monitoring so that accurate and complete data can be collected or from using a dietary metric that measures adherence to sustainable and healthy diets^(50)^.

It is also possible that the null findings in our study were a result of the relatively short follow-up period, which may not have been sufficient for the lifestyle arm to embed dietary changes introduced during the intervention. Likewise, the FFQ used in this study was not designed to measure short-term dietary changes and is typically used to measure habitual dietary patterns over time. Finally, the possible impact of multiple testing cannot be overlooked, particularly given that findings were weakened when adjusting for multiple testing. However, the findings remained in the same direction, and given the low sample size, we believe they provide important clues that can be evaluated in future research. Future studies might expand on ours by measuring other environmental impacts such as water use, acidification and land clearing, which are not considered in the GWP* variable, as well as unaccounted for variables such as alterations in transportation modes and reductions in environmental pollutants.

The findings of this study highlight the broader ecological implications of dietary choices, emphasising the need for a comprehensive understanding of the environmental impact of different dietary interventions and the challenges of balancing human health considerations with planetary health impacts. In sum, future iterations of lifestyle therapy aimed at improving mental health symptoms could be easily adapted to include education on the planetary impacts of different food groups, particularly food classifications with a higher environmental impact and that may also adversely impact human health, such as discretionary foods and drinks and UPF. Whilst meat has been consistently shown to be one of the biggest contributors to the environmental impact of food systems, the human health impacts of reducing or removing this entirely require further investigation and nuanced local recommendations developed to promote sustainable animal-based food choices.

## Supporting information

Turner et al. supplementary material Turner et al. supplementary material

## Supplementary material

For supplementary material/s referred to in this article, please visit https://doi.org/10.1017/S0007114525103942.
