# Supplementary material for: Environmental co-benefits of a Mediterranean-style dietary intervention for reducing depressive symptoms in adults: results from the Curbing Anxiety and Depression using Lifestyle Medicine randomised controlled trial
Source: Br J Nutr. 2025 Jul 18;134(2):115–23. doi: 10.1017/S0007114525103942 (PMC12433746; doi:10.1017/S0007114525103942)
Supplement: Turner et al. supplementary material [file S0007114525103942sup001.docx]

Supplementary Table 1: Food items from the DQES, their NHMRC group and subgroup, NOVA category, and GWP* value per kilogram of food intake.

| **Food Item** | **NHMRC group** | **Subgroup** | **NOVA category** | **GWP* value** |
| --- | --- | --- | --- | --- |
| All other cheeses | Dairy and alternatives | Dairy | Processed food | 6.28 |
| Full cream milk | Dairy and alternatives | Dairy | Unprocessed | 1.23 |
| Reduced fat milk | Dairy and alternatives | Dairy | Unprocessed | 0.86 |
| Ricotta or cottage cheese | Dairy and alternatives | Dairy | Processed food | 6.28 |
| Skim milk | Dairy and alternatives | Dairy | Unprocessed | 0.86 |
| Yogurt | Dairy and alternatives | Dairy | Ultra-processed | 3.27 |
| Rice milk | Dairy and alternatives | Dairy alternative | Processed food | -0.03 |
| Soy milk | Dairy and alternatives | Dairy alternative | Ultra-processed | 0.12 |
| Bacon | Discretionary | Animal source | Processed food | 4.58 |
| Butter | Discretionary | Animal source | Processed culinary ingredient | 8.37 |
| Butter dairy blend on vegetables | Discretionary | Animal source | Processed culinary ingredient | 0.6275 |
| Butter margarine blends | Discretionary | Animal source | Processed culinary ingredient | 5.474 |
| Chocolate | Discretionary | Animal source | Ultra-processed | 3.91 |
| Flavoured milk | Discretionary | Animal source | Ultra-processed | 1.3 |
| Fried fish | Discretionary | Animal source | Unprocessed | 3.49 |
| Ice cream | Discretionary | Animal source | Ultra-processed | 2.32 |
| Mayonnaise | Discretionary | Animal source | Ultra-processed | 1.28 |
| Pastries with cheese | Discretionary | Animal source | Ultra-processed | 3.35 |
| Pastries with meat | Discretionary | Animal source | Ultra-processed | 2.64 |
| Pizza | Discretionary | Animal source | Unprocessed | 0.64 |
| Processed Meat | Discretionary | Animal source | Ultra-processed | 5.39 |
| Sausages | Discretionary | Animal source | Ultra-processed | 3.61 |
| Cream or sour cream | Discretionary | Animal source | Processed culinary ingredient | 4.68 |
| Cakes or sweet pastries | Discretionary | Other source | Ultra-processed | 1.34 |
| Coleslaw | Discretionary | Other source | Unprocessed | 0.22 |
| Corn chips etc | Discretionary | Other source | Ultra-processed | 2.98 |
| Diet soft drink | Discretionary | Other source | Ultra-processed | 0.1 |
| Heavy beer | Discretionary | Other source | Processed food | 0.27 |
| Jam etc | Discretionary | Other source | Processed food | 1.63 |
| Light beer | Discretionary | Other source | Processed food | 0.27 |
| Other confectionery | Discretionary | Other source | Ultra-processed | 0.93 |
| Potato cooked in fat | Discretionary | Other source | Ultra-processed | 0.344 |
| Red wine | Discretionary | Other source | Processed food | 0.96 |
| Soft drink | Discretionary | Other source | Ultra-processed | 0.18 |
| Spirits | Discretionary | Other source | Ultra-processed | 0.25 |
| Spirits – premix | Discretionary | Other source | Ultra-processed | 1.62 |
| Sugar | Discretionary | Other source | Processed culinary ingredient | 0.72 |
| Sugary cereals | Discretionary | Other source | Ultra-processed | 0.305 |
| sweet biscuits | Discretionary | Other source | Ultra-processed | 1.66 |
| Tomato sauce or ketchup | Discretionary | Other source | Ultra-processed | 0.93 |
| Vegemite etc | Discretionary | Other source | Ultra-processed | 1.13 |
| White wine | Discretionary | Other source | Processed food | 0.96 |
| Canola margarine | Unsaturated spreads and oils | Vegetal fats | Ultra-processed | 1.13 |
| Canola oil | Unsaturated spreads and oils | Vegetal fats | Processed culinary ingredient | 1.16 |
| Cholesterol lowering margarine | Unsaturated spreads and oils | Vegetal fats | Ultra-processed | 1.13 |
| Low-calorie, low-fat salad dressing | Unsaturated spreads and oils | Vegetal fats | Ultra-processed | 1.3947 |
| Margarine on vegetables | Unsaturated spreads and oils | Vegetal fats | Ultra-processed | 0.27 |
| Oil and vinegar salad dressing | Unsaturated spreads and oils | Vegetal fats | Ultra-processed | 1.81 |
| Oil on vegetables | Unsaturated spreads and oils | Vegetal fats | Processed culinary ingredient | 0.37 |
| Olive oil | Unsaturated spreads and oils | Vegetal fats | Processed culinary ingredient | 3.26 |
| Olive oil margarine | Unsaturated spreads and oils | Vegetal fats | Ultra-processed | 1.45 |
| Olive Oil on Bread | Unsaturated spreads and oils | Vegetal fats | Processed culinary ingredient | 0.91 |
| Poly margarine | Unsaturated spreads and oils | Vegetal fats | Ultra-processed | 1.13 |
| Vegetable oil | Unsaturated spreads and oils | Vegetal fats | Processed culinary ingredient | 1.16 |
| Apples | Fruit | Fruit | Unprocessed | 0.3 |
| Apricots | Fruit | Fruit | Unprocessed | 0.45 |
| Bananas | Fruit | Fruit | Unprocessed | 0.41 |
| Berries | Fruit | Fruit | Unprocessed | 0.45 |
| Cantaloupe | Fruit | Fruit | Unprocessed | 0.45 |
| Cherries | Fruit | Fruit | Unprocessed | 0.45 |
| Dried apricots | Fruit | Fruit | Unprocessed | 2.43 |
| Figs | Fruit | Fruit | Unprocessed | 0.45 |
| Grapes | Fruit | Fruit | Unprocessed | 0.37 |
| Kiwi Fruit | Fruit | Fruit | Unprocessed | 0.45 |
| Mango or pawpaw | Fruit | Fruit | Unprocessed | 0.64 |
| Olives | Fruit | Fruit | Processed food | 0.45 |
| Orange juice | Fruit | Fruit | Unprocessed | 0.71 |
| Oranges | Fruit | Fruit | Unprocessed | 0.41 |
| Other dried fruit | Fruit | Fruit | Unprocessed | 1.86 |
| Other fruit juice | Fruit | Fruit | Ultra-processed | 1.12 |
| Peaches or nectarines | Fruit | Fruit | Unprocessed | 0.45 |
| Pears | Fruit | Fruit | Unprocessed | 0.34 |
| Pineapple | Fruit | Fruit | Unprocessed | 0.41 |
| Plums | Fruit | Fruit | Unprocessed | 0.45 |
| Strawberries | Fruit | Fruit | Unprocessed | 0.45 |
| Tinned fruit | Fruit | Fruit | Processed food | 0.49 |
| Watermelon | Fruit | Fruit | Unprocessed | 0.45 |
| Bran cereals | Grains | Grains | Ultra-processed | 0.52 |
| Breakfast cereal Avg | Grains | Grains | Ultra-processed | 0.49 |
| Cornflakes | Grains | Grains | Ultra-processed | 0.54 |
| Crackers or dry biscuits not wholemeal | Grains | Grains | Ultra-processed | 0.87 |
| Gluten free bread | Grains | Grains | Ultra-processed | -0.91 |
| Hi fibre white bread | Grains | Grains | Ultra-processed | 0.64 |
| Mixed grain cereals | Grains | Grains | Ultra-processed | 0.65 |
| Muesli toasted | Grains | Grains | Unprocessed | 1.08 |
| Muesli untoasted | Grains | Grains | Unprocessed | 1.08 |
| Multi grain bread | Grains | Grains | Ultra-processed | 0.64 |
| Nutri Grain | Grains | Grains | Ultra-processed | 0.65 |
| Pasta or noodles | Grains | Grains | Unprocessed | 0.8 |
| Porridge Avg | Grains | Grains | Unprocessed | 0.67 |
| Rice | Grains | Grains | Unprocessed | -0.86 |
| Rice cereals | Grains | Grains | Ultra-processed | -0.54 |
| Rye Bread | Grains | Grains | Ultra-processed | 0.64 |
| Soy and linseed bread | Grains | Grains | Ultra-processed | 0.64 |
| Special K | Grains | Grains | Ultra-processed | 0.43 |
| Wheat biscuits flakes | Grains | Grains | Ultra-processed | 0.43 |
| white Bread | Grains | Grains | Ultra-processed | 0.64 |
| Wholemeal Bread | Grains | Grains | Ultra-processed | 0.66 |
| Wholemeal crackers or dry biscuits | Grains | Grains | Ultra-processed | 0.8 |
| Eggs | Lean meats and alternatives | Eggs | Unprocessed | 1.51 |
| Other seafood | Lean meats and alternatives | Fish/seafood | Unprocessed | 3.49 |
| Steamed fish | Lean meats and alternatives | Fish/seafood | Unprocessed | 3.49 |
| Tinned fish | Lean meats and alternatives | Fish/seafood | Processed food | 3.49 |
| Baked beans | Lean meats and alternatives | Meat alternatives | Ultra-processed | 0.33 |
| Dried beans, peas, chickpeas | Lean meats and alternatives | Meat alternatives | Unprocessed | 0.33 |
| Other nuts | Lean meats and alternatives | Meat alternatives | Unprocessed | 2.69 |
| Peanuts or peanut butter | Lean meats and alternatives | Meat alternatives | Unprocessed | 0.89 |
| Chicken | Lean meats and alternatives | Monogastric meat | Unprocessed | 2.66 |
| Pork | Lean meats and alternatives | Monogastric meat | Unprocessed | 3.78 |
| Beef or veal | Lean meats and alternatives | Ruminant meat | Unprocessed | 16.68 |
| Lamb | Lean meats and alternatives | Ruminant meat | Unprocessed | -4.8 |
| Asian greens | Vegetables | Vegetables | Unprocessed | 0.22 |
| Asparagus | Vegetables | Vegetables | Unprocessed | 0.22 |
| Avocado | Vegetables | Vegetables | Unprocessed | 0.6 |
| Beetroot | Vegetables | Vegetables | Unprocessed | 0.22 |
| Broccoli | Vegetables | Vegetables | Unprocessed | 0.22 |
| Brussels sprouts | Vegetables | Vegetables | Unprocessed | 0.22 |
| Canned tomatoes | Vegetables | Vegetables | Unprocessed | 0.22 |
| Capsicum | Vegetables | Vegetables | Unprocessed | 0.22 |
| Carrots | Vegetables | Vegetables | Unprocessed | 0.31 |
| Cauliflower | Vegetables | Vegetables | Unprocessed | 0.22 |
| Celery | Vegetables | Vegetables | Unprocessed | 0.22 |
| Cucumber | Vegetables | Vegetables | Unprocessed | 0.22 |
| Eggplant | Vegetables | Vegetables | Unprocessed | 0.22 |
| Fresh tomatoes | Vegetables | Vegetables | Unprocessed | 0.22 |
| Garlic | Vegetables | Vegetables | Unprocessed | 0.2 |
| Green beans | Vegetables | Vegetables | Unprocessed | 0.33 |
| Green peas | Vegetables | Vegetables | Unprocessed | 0.33 |
| Iceberg lettuce | Vegetables | Vegetables | Unprocessed | 0.22 |
| Mushrooms | Vegetables | Vegetables | Unprocessed | 0.22 |
| Onion or leeks | Vegetables | Vegetables | Unprocessed | 0.2 |
| Other cooked leafy vegetables | Vegetables | Vegetables | Unprocessed | 0.22 |
| Other lettuce and salad leaves | Vegetables | Vegetables | Unprocessed | 0.22 |
| Potato cooked without fat | Vegetables | Vegetables | Unprocessed | 0.31 |
| Pumpkin | Vegetables | Vegetables | Unprocessed | 0.22 |
| Sweet corn | Vegetables | Vegetables | Unprocessed | 0.22 |
| Sweet Potato | Vegetables | Vegetables | Unprocessed | 0.31 |
| Tomato products | Vegetables | Vegetables | Processed food | 0.22 |
| Zucchini or squash | Vegetables | Vegetables | Unprocessed | 0.22 |
| Coffee | Water-based beverage | Water-based beverage | Unprocessed | 7.08 |
| Coffee substitute | Water-based beverage | Water-based beverage | Ultra-processed | 0.39 |
| Herbal tea | Water-based beverage | Water-based beverage | Unprocessed | 1.25 |
| Tea | Water-based beverage | Water-based beverage | Unprocessed | 1.25 |
| Water | Water-based beverage | Water-based beverage | Unprocessed | 0.04 |

Supplementary Table 2: Median (quartile 1 to quartile 3) baseline and 8-week dietary intake (grams per day); median difference and 95% confidence intervals (baseline to 8 weeks)

|  | **Psychotherapy (n=64)** | | | **Lifestyle Therapy (n=71)** | | |
| --- | --- | --- | --- | --- | --- | --- |
|  | Baseline median  (q1-q3) | 8 Weeks median  (q1-q3) | Median difference† (95% CI) | Baseline median (q1-q3) | 8 Weeks median  (q1-q3) | Median difference† (95% CI) |
| **NHMRC/ADG Groups** | | | | | | |
| Total energy intake (kJ/day) | 7960.6  (6798.4-9670.8) | 7796.6  (6697.9-8702.7) | -397.44  (-1193.87, 460.18) | 8632.3  (7351.8-10680.2) | 8919.6  (7396.2-9989.4) | -87.08  (-1038.58, 759.16) |
| Dairy and Alternatives | 259.8  (150.4-519.9) | 284.4  (168.3-391.9) | 16.55  (-43.50, 91.10) | 275.3  (166.4-508.0) | 310.6  (232.6-502.1) | 37.80  (-20.80, 97.70) |
| Dairy | 177.2  (54.9-366.8) | 191.4  (52.5-337.7) | -11.55  (-83.40, 46.20) | 176.3  (92.4-352.4) | 211.4  (129.5-362.5) | 26.20  (-28.80, 95.90) |
| Dairy Alternative | 0.0  (0.0-62.5) | 0.0  (0.0-83.3) | 0.00  (0.00, 0.00) | 0.0  (0.0-125.0) | 0.0  (0.0-62.5) | 0.00  (0.00, 0.00) |
| Discretionary | 411.4  (168.5-802.0) | 338.6  (219.5-680.6) | -16.80  (-153.60, 102.90) | 430.4  (263.6-667.7) | 307.8  (167.2-501.5) | ***-108.30***  ***(-220.40, -8.00)*** |
| Animal Source | 71.1  (46.6-102.2) | 67.8  (42.9-97.7) | -4.10  (-19.30, 11.80) | 74.8  (50.8-131.6) | 62.7  (40.3-93.2) | ***-19.70***  ***(-37.10, -3.70)*** |
| Other | 321.2  (109.9-621.9) | 287.9  (133.0-603.5) | -23.45  (-156.10, 86.60) | 326.6  (212.0-533.9) | 257.3  (110.6-387.6) | -88.80  (-184.70, 1.80) |
| Unsaturated spreads and oils | 20.3  (14.7-32.2) | 20.2  (16.4-29.4) | 1.15  (-3.10, 5.10) | 22.9  (15.5-40.0) | 30.5  (18.3-37.0) | 2.90  (-2.60, 8.80) |
| Fruit | 178.4  (98.3-262.2) | 193.4  (97.3-266.1) | 3.80  (-39.10, 49.90) | 185.2  (109.8-269.8) | 215.4  (152.7-282.7) | 13.60  (-31.90, 59.30) |
| Grains | 183.3  (123.6-243.0) | 174.2  (138.8-235.9) | 5.10  (-27.30, 38.70) | 199.6  (151.8-240.8) | 205.1  (152.3-269.8) | 7.40  (-27.10, 44.60) |
| Lean Meats and Alternatives | 220.0  (182.2-290.4) | 219.3  (165.8-282.7) | -9.10  (-42.90, 27.90) | 207.8  (159.9-313.7) | 262.6  (193.9-321.3) | 27.50  (-12.80, 69.70) |
| Eggs | 17.1  (11.4-45.7) | 17.1  (17.1-45.7) | 0.00  (0.00, 0.00) | 17.1  (17.1-45.7) | 17.1  (17.1-45.7) | 0.00  (0.00, 0.00) |
| ***Fish/Seafood*** | 20.4  (9.3-34.3) | 19.1  (7.9-36.6) | -0.40  (-7.90, 5.70) | 15.7  (7.9-22.0) | 27.6  (15.5-58.7) | ***9.50***  ***(2.40, 18.00)*** |
| Meat Alternatives | 49.6  (24.0-106.4) | 52.9  (25.7-93.6) | 2.00  (-16.20, 21.70) | 44.9  (28.9-89.7) | 76.8  (46.4-114.6) | 20.00  (-0.20, 41.00) |
| Monogastric Meat | 55.6  (30.5-95.0) | 48.8  (24.3-85.0) | -3.65  (-21.60, 9.50) | 52.3  (29.6-81.5) | 51.1  (27.1-84.2) | 0.00  (-15.20, 12.30) |
| Ruminant Meat | 42.6  (14.9-63.3) | 33.5  (8.0-72.4) | 0.00  (-17.00, 9.10) | 42.6  (14.9-85.2) | 49.0  (18.6-85.2) | 0.60  (-12.80, 18.60) |
| ***Vegetables*** | 224.8  (138.1-351.0) | 253.1  (185.8-322.7) | 21.25  (-30.30, 71.00) | 186.4  (137.0-290.7) | 276.4  (202.5-351.1) | ***69.40***  ***(17.20, 117.90)*** |
| Water-based beverages | 1724.2  (1224.0-2306.0) | 1810.5  (1336.2-2411.2) | 44.80  (-231.80, 342.80) | 1710.5  (1304.0-2230.0) | 1882.1  (1175.4-2255.0) | 32.00  (-236.40, 314.40) |
| **NOVA Groups** | | | | | | |
| Un-/minimally processed | 2897.6  (2036.3-3359.3) | 2747.0  (2206.2-3545.2) | 66.40  (-316.30, 472.40) | 2760.3  (2180.7-3137.8) | 2961.2  (2297.4-3522.3) | 125.60  (-208.00, 484.30) |
| Processed culinary ingredients | 26.3  (17.7-38.8) | 27.0  (19.3-35.1) | 0.70  (-4.60, 5.80) | 33.6  (20.4-45.6) | 32.5  (24.9-48.9) | 1.60  (-5.10, 9.60) |
| Processed foods | 146.0  (73.0-311.6) | 150.0  (60.2-300.5) | 0.75  (-41.90, 46.90) | 189.8  (65.9-375.3) | 150.3  (97.9-346.4) | -10.00  (-66.90, 42.50) |
| Ultra-processed foods | 385.2  (185.5-800.6) | 313.0  (229.8-610.9) | -24.25  (-128.80, 99.30) | 431.5  (255.5-612.2) | 349.2  (207.6-588.8) | -63.60  (-159.50, 29.40) |

†Hodges-Lehmann median difference

Supplementary Table 3: Results from the imputed per-protocol^†^ and intention-to-treat^‡^ models showing association of treatment allocation with percentage change in GWP* score between baseline and 8-weeks follow-up.

|  | | **Unadjusted** | | | | **Adjusted§** | | | |
| --- | --- | --- | --- | --- | --- | --- | --- | --- | --- |
|  |  | β | L95CI | U95CI | p-value | β | L95CI | U95CI | p-value |
| Total GWP* | ITT | 5.03 | -9.26 | 19.32 | 0.245 | 5.53 | -8.21 | 19.27 | 0.215 |
|  | PP | 5.64 | -9.84 | 21.12 | 0.237 | 6.16 | -9.63 | 21.95 | 0.222 |
| **NHMRC/ADG Groups** | | | | | | | | | |
| Dairy and Alternatives | ITT | 4.42 | -21.96 | 30.80 | 0.371 | 9.43 | -15.79 | 34.66 | 0.231 |
|  | PP | 9.16 | -19.39 | 37.72 | 0.265 | 17.11 | -8.89 | 43.12 | 0.099 |
| Dairy | ITT | -0.43 | -42.92 | 42.05 | 0.492 | 3.29 | -38.66 | 45.23 | 0.439 |
|  | PP | 1.60 | -46.01 | 49.20 | 0.474 | 7.61 | -39.83 | 55.04 | 0.377 |
| Dairy Alternative | ITT | 7.68 | -85.46 | 100.82 | 0.436 | 20.39 | -47.70 | 88.49 | 0.278 |
|  | PP | 12.40 | -98.64 | 123.44 | 0.413 | 32.24 | 51.31 | 117.80 | 0.220 |
| Discretionary | ITT | -6.79 | -21.73 | 8.15 | 0.186 | -7.74 | -21.93 | 6.46 | 0.143 |
|  | PP | -8.31 | -25.13 | 8.52 | 0.166 | -9.41 | -25.38 | 6.57 | 0.124 |
| Animal Source | ITT | -5.89 | -25.44 | 13.66 | 0.277 | -6.27 | -25.28 | 12.73 | 0.259 |
|  | PP | -6.83 | -29.16 | 15.49 | 0.274 | -6.99 | -28.56 | 14.59 | 0.263 |
| Other | ITT | -1.22 | -23.10 | 20.65 | 0.456 | -3.49 | 24.60 | 17.62 | 0.373 |
|  | PP | -2.37 | -26.65 | 21.91 | 0.424 | -5.77 | -29.90 | 18.36 | 0.319 |
| Fat | ITT | 12.84 | -12.28 | 37.96 | 0.157 | 12.94 | -11.63 | 37.51 | 0.150 |
|  | PP | 17.07 | -8.91 | 43.04 | 0.099 | 17.39 | -9.19 | 43.97 | 0.100 |
| Fruit | ITT | 3.32 | -20.65 | 27.29 | 0.393 | 6.12 | -17.20 | 29.45 | 0.303 |
|  | PP | 2.40 | -23.01 | 27.81 | 0.427 | 6.22 | -19.60 | 32.04 | 0.318 |
| Grains | ITT | 33.96 | -238.45 | 306.38 | 0.403 | 33.47 | -230.78 | 297.72 | 0.402 |
|  | PP | 34.50 | -264.01 | 333.01 | 0.410 | 30.74 | -272.48 | 333.97 | 0.421 |
| Lean Meats and Alternatives | ITT | 9.34 | -15.01 | 33.69 | 0.226 | 5.30 | -18.97 | 29.58 | 0.334 |
|  | PP | 12.20 | -14.81 | 39.22 | 0.188 | 5.49 | -22.04 | 33.01 | 0.348 |
| Eggs | ITT | 9.51 | -11.39 | 30.41 | 0.186 | 8.73 | -11.81 | 29.26 | 0.202 |
|  | PP | 13.50 | -11.86 | 38.85 | 0.149 | 12.20 | -12.84 | 37.24 | 0.170 |
| Fish/Seafood | ITT | 28.80 | -33.01 | 90.60 | 0.180 | 25.65 | -33.84 | 85.14 | 0.199 |
|  | PP | 41.05 | -22.02 | 104.12 | 0.101 | 38.45 | -23.56 | 100.47 | 0.112 |
| Meat Alternatives | ITT | 35.26 | -18.26 | 88.79 | 0.098 | 27.73 | -24.18 | 79.64 | 0.147 |
|  | PP | 51.45 | -6.53 | 109.43 | 0.041 | 41.85 | -15.62 | 99.32 | 0.077 |
| Monogastric Meat | ITT | 4.27 | -11.19 | 19.73 | 0.294 | 2.55 | -12.52 | 17.61 | 0.370 |
|  | PP | 6.08 | -9.36 | 21.51 | 0.220 | 4.32 | -11.37 | 20.01 | 0.294 |
| Ruminant Meat | ITT | 11.06 | -65.10 | 87.22 | 0.388 | -3.55 | -80.96 | 73.86 | 0.464 |
|  | PP | 26.53 | -57.50 | 110.55 | 0.268 | 11.26 | -77.30 | 99.83 | 0.402 |
| Vegetables | ITT | 5.79 | -14.68 | 26.27 | 0.289 | 4.37 | -15.76 | 24.49 | 0.335 |
|  | PP | 6.87 | -16.86 | 30.60 | 0.285 | 4.99 | -18.69 | 28.67 | 0.340 |
| Water-based beverages | ITT | 0.71 | -24.05 | 25.47 | 0.478 | 0.08 | -24.51 | 24.67 | 0.498 |
|  | PP | 1.20 | -28.02 | 30.42 | 0.468 | -0.07 | -29.17 | 29.04 | 0.498 |
| **Nova UPF Groups** | | | | | | | | | |
| Unprocessed/minimally processed | ITT | 5.30 | -8.44 | 19.04 | 0.225 | 5.76 | -7.65 | 19.17 | 0.200 |
|  | PP | 5.90 | -9.24 | 21.04 | 0.222 | 6.25 | -9.29 | 21.80 | 0.215 |
| Processed culinary ingredients | ITT | 4.18 | -18.80 | 27.17 | 0.361 | 4.04 | -18.70 | 26.78 | 0.364 |
|  | PP | 6.55 | -18.93 | 32.03 | 0.307 | 6.65 | -19.44 | 32.74 | 0.309 |
| Processed foods | ITT | 6.67 | -10.86 | 2419.00 | 0.227 | 7.31 | -9.92 | 24.54 | 0.202 |
|  | PP | 8.57 | -10.55 | 27.69 | 0.190 | 9.47 | -9.71 | 28.65 | 0.166 |
| Ultra-processed foods | ITT | -5.81 | -20.88 | 9.27 | 0.225 | -6.21 | -19.49 | 7.07 | 0.180 |
|  | PP | -7.17 | -23.93 | 9.58 | 0.200 | -7.57 | -22.25 | 7.10 | 0.156 |

^†^PP=Per-protocol analysis, based on all 135 participants who completed the intervention, with missing values imputed using multiple imputation.
^‡^ITT=intention to treat analysis, based on imputation of all 182 randomised participants
^§^Adjusted for baseline GWP*, age, sex, and energy intake using Willet’s residual method.
